# Serum C peptide level and renal function in diabetes mellitus

**DOI:** 10.4103/0971-4065.62093

**Published:** 2010

**Authors:** M. N. Chowta, P. M. Adhikari, N. K. Chowta, A. K. Shenoy, S. D'Souza

**Affiliations:** Department of Pharmacolgy, Kasturba Medical College, Mangalore, India; 1 Department of Medicine, Kasturba Medical College, Mangalore, India; 2 Department of Ophthalmology, Kasturba Medical College, Mangalore, India

**Keywords:** C peptide, creatinine clearance, microalbuminuria

## Abstract

C peptide is an active peptide hormone with potentially important physiological effects. C peptide has the capacity to diminish glomerular hyperfiltration and reduce urinary albumin excretion in both experimental and human type 1 diabetes. The present study is aimed at correlating the serum C peptide level with that of renal clearance, urinary albumin excretion and duration of diabetes. This is a prospective cross sectional study. Patients with diagnosis of type 2 diabetes mellitus were evaluated for their baseline clinical and laboratory profile. Both males and females above the age of 18 years were included in the study. The laboratory investigations include fasting serum C peptide, HbA_1C_, serum creatinine, blood urea nitrogen, urine albumin and creatinine. Creatinine clearance was calculated using modification of diet in renal disease formula from serum creatinine value. A total of 168 patients were included in the study, among them 90 were females (53.57%) and 78 males (46.43%). Mean age of the patients was 57.64 years. Pearson correlation test showed negative correlation of serum C peptide level with creatinine clearance, though statistically not significant. Negative correlation was also seen between serum C peptide, and urine albumin, urine albumin creatinine ratio, HbA_1C_ and duration of diabetes. Mean urine albumin was higher in patients with subnormal C peptide level. Duration of disease was more in patients with lower serum C peptide level. The study has shown weak association of serum C peptide level with microalbuminuria and creatinine clearance. Risk of albuminuria is more in patients with low serum C peptide level.

## Introduction

C peptide is a polypeptide with a molecular weight of 3600, containing 31 amino acids. In insulin bio-synthesis, C-peptide is cleaved from pro-insulin, stored in secretory granules, and eventually released into the bloodstream in amounts equimolar with those of insulin. C-peptide has an essential function in the synthesis of insulin in that it links the A and B chains in a manner that allows correct folding and inter-chain disulfide bond formation.[1] The kidney has been suggested as the main organ for the degradation of C-peptide. Half-life of C peptide in circulation is 2–5 times longer than insulin.[2] C peptide is the more reliable indicator of insulin secretion than insulin itself. Furthermore, the concentration of C peptide is not affected by interference from insulin antibodies often present in patients receiving insulin therapy. Indication for C peptide measurement recently expanded to permit evaluation of insulin dependence in maturity onset diabetes mellitus. It is generally accepted that C-peptide possesses little or no biological activity and has no other role than its participation in insulin synthesis, a role that is emphasized by its name, “connecting peptide.” The currently available information establishes that C-peptide is not as biologically inert as previously believed. Instead, it now emerges as an active peptide hormone with potentially important physiological effects. Even though C-peptide is formed from pro-insulin and co-secreted with insulin, we should consider the possibility that C-peptide is a separate entity with biochemical and physiological characteristics that are different from those of insulin.[3,4] C-peptide has the capacity to diminish glomerular hyperfiltration and reduce urinary albumin excretion in both experimental and type 1 diabetes.[5,6] The present study is aimed at correlating the serum C peptide level with that of renal clearance, urinary albumin excretion and duration of diabetes.

## Materials and Methods

This is a prospective cross sectional study. Patients with diagnosis of type 2 diabetes mellitus, including newly diagnosed cases, were evaluated for their baseline clinical and laboratory profile. Data was collected from patients screened for participation in clinical trials on type 2 diabetes mellitus. All the trials had been approved by Institution Ethics Committee and informed consent has been taken from each patient who was screened. Both males and females above the age of 18 years were included in the study. Detailed history, including duration of the disease, was collected. All patients underwent thorough physical examination. Details regarding anti diabetic medication and concomitant diseases were also collected. Body weight and body mass index (BMI) were measured in all patients.

Laboratory investigations done included fasting serum C peptide, HbA_1C_, serum creatinine, blood urea nitrogen, urine albumin and creatinine. Creatinine clearance was calculated from serum creatinine vale using MDRD formula.

### Statistical analysis

Data was analyzed using student “*t*” test to compare the values between different category patients. Correlation analysis was done by using Pearson correlation. A *P* value <0.05 were considered significant.

## Results

A total of 168 patients were included in the study, 90 females (53.57%) and 78 males (46.43%). The mean age of the patients was 57.64 years. Demographic data of patients were as shown in Table 1. Pearson correlation test showed negative correlation of serum C peptide level with creatinine clearance (r = -0.123, *P*>0.05), though statistically not significant. Negative correlation was also seen with serum C peptide vs urine albumin level (r = -0.059, *P*>0.05), vs. urine albumin creatinine ratio (r = -0.015, *P*>0.05), vs. HbA_1C_ (r = -0.207, *P*>0.05) and vs. duration of diabetes (r = -0.171, *P*>0.05) [Table 2]. Serum C peptide was normal (category 1) in 130 patients (77.38%), among them 69 were females (53.08%) and 61 were males (46.92%). Serum C peptide was above normal (category 2) in 29 patients (17.326), among them 17 were females (53.08%) and 12 were males (41.38%). Serum C peptide below normal (category 3) in nine patients (5.36%), among them four were female (44.44%) and five male (55.56%) [Table 3]. Mean creatinine clearance in category 1 patients was 81.29 ± 1.6 ml minute, in category 2 patients was 78.62 ± 4.51 ml/minute and in category 3 patients was 81.22 ± 5.04/minute. Though the creatinine clearance was less in patients with elevated C peptide, it did not show any statistical significance. Mean urine albumin in category 1 patients was 19.31 ± 3.96 mg/L, in category 2 patients was 21.31 ± 8.6 mg/L and in category 3 patients was 43.43 ± 31.58 mg/L. The value was higher in patients with below normal C peptide level, though statistical significance was not observed. Duration of disease more in patients with below normal serum C peptide level compared to other two categories of patients. BMI was lower in patients with low serum peptide and comparatively higher in patients with elevated serum C peptide level. HbA_1C_ was also higher in patients with low serum C peptide level compared to other two groups. All the patients were on oral hypoglycaemic agents either as monotherapy or two drug combination.

**Table 1:** Demographic characteristics of the patients

Characteristics	Value
Age (years)	57.64 ± 0.91
Males (n (%))	78 (46.43)
Females (n (%)	90 (53.57)
Body mass index (mg/m^2^)	25.34 ± 0.3
Duration of disease (years)	4.26 ± 0.45
HbA_1C_ (%)	8.14 ± 0.14
Serum creatinine (micromole/L)	80.06 ± 1.31
Creatinine clearance (ml/min)	80.91 ± 1.45
Blood urea nitrogen (mmole/min)	3.91 ± 0.1
Urine albumin (mg/L)	20.58 ± 3.72
Serum C peptide (nmol/L)	0.97 ± 0.05

All values are expressed as mean ± SEM

**Table 2:** Correlation of serum C peptide level with other parameters

C peptide vs	Correlation coefficient (r value)	*P* value
Creatinine clearance	-0.123	>0.05
Urine albumin	-0.059	>0.05
Duration of disease	-0.171	>0.05
HbA_1C_	-0.207	>0.05

Pearson correlation test

**Table 3:** Comparison of baseline characteristics among different categories of patients

Characteristics	Category 1	Category 2	Category 3
Age (years)	57.47 ± 1.1	59.41 ± 2.25	53 ± 5.63
Males (n (%)	61 (46.92)	12 (41.38)	5 (55.56)
Females (n (%)	69 (53.08)	17 (58.62)	4 (44.44)
Body mass index (mg/m^2^)	25.25 ± 0.33	26.17 ± 0.73	23.71 ± 1.5
Duration of disease (years)	4.13 ± 0.5	4.31 ± 1.28	5.38 ± 1.83
HbA_1C_ (%)	8.02 ± 0.15	8.1 ± 0.31	9.21 ± 1.03
Serum creatinine (micromole/L)	79.46 ± .38	84.4 ± 4.26	78.57 ± 3.37
Creatinine clearance (ml/min)	81.29 ± 1.6	78.62 ± 4.51	81.22 ± 5.04
Blood urea nitrogen (mmole/min)	3.93 ± 0.11	3.8 ± 0.25	4.12 ± 0.24
Urine albumin (mg/L)	19.31 ± 3.96	21.31 ± 8.6	43.43 ± 31.58
Serum C peptide(nmol/L)	0.8 ± 0.02	1.91 ± 0.14	0.23 ± 0.03

All values are expressed as mean ± SEM; Category 1 = Normal serum C peptide, Category 2 = Elevated serum C peptide, Category 3 = Low serum C peptide

## Discussion

After the discovery of the mode of insulin biosynthesis, several early studies addressed the question of possible physiological effects of C-peptide. Insulin-like effects on blood glucose levels and glucose disposal after glucose loading were looked for but not found.[7,8] Recently, new data have been presented demonstrating specific binding of C-peptide to cell surfaces in a manner that suggests the presence of G protein-coupled membrane receptors. C-peptide may thereby stimulate specific intracellular processes, influencing renal and nerve function in C-peptide-deficient type 1 diabetes patients.[3]

In this study we found weak association of serum C peptide with renal parameters and duration of diabetes. Serum C peptide level was negatively correlated with creatinine clearance, urine albumin excretion and urine albumin creatinine ratio. Patients with type 1 diabetes frequently develop glomerular hyperfiltration early in the course of their disorder.[9] Adequate insulin therapy does not correct this phenomenon.[10] In contrast, patients with type 2 diabetes, in whom insulin and C-peptide levels are within or above the normal range, do not show glomerular hyperfiltration or hypertrophy.[11,12] The mechanism underlying the beneficial effect of C-peptide on renal function in diabetes is not known. However, it is possible that C-peptide may have exerted a direct effect on the glomerular handling of albumin, as suggested by the studies of renal function in animals with experimental diabetes. The influence of C-peptide on glomerular hyperfiltration and renal protein leakage has been examined in streptozotocin diabetic rats.[5] Administration of C-peptide for 90 minute was accompanied by diminished glomerular hyperfiltration and a marked reduction in protein leakage compared with diabetic control animals. C-peptide has the capacity to stimulate both renal Na^+^-K^+^-ATPase and eNOS (endothelial nitric oxide synthase). Both of these enzyme systems are known to show attenuated activities in type 1 diabetes, particularly in renal and nerve tissue.[13,14] C-peptide can influence glomerular membrane permeability and transport, as well as regional blood flow of the kidney. There is now evidence to indicate that replacement of C-peptide in type 1 diabetes is accompanied by improved renal function, as evidenced by correction of glomerular hyperfiltration and diminished urinary albumin excretion, and amelioration of nerve dysfunction.[15]

The present study has shown the negative correlation of serum C peptide level with duration of disease, which may be indicating progressive beta cell failure. HbA_1C_ also showed negative correlation with serum C peptide level suggesting poor glycaemic control in patients with low serum C peptide level and indicating the need for insulin therapy. C peptide is the more stable indicator of insulin secretion than insulin and its measurement is important in the evaluation of insulin dependency even in maturity onset diabetes mellitus.

Our study also has shown negative correlation of C peptide value with urine albumin level, though statistically not significant. C peptide diminishes glomerular hyperfiltration and causes reduction in protein leakage.[5] The number of patients with below normal C peptide was small in our study, which may be the reason for absence of significant correlation between C peptide level and urine albumin excretion rate.

Limitations of our study deserve comment. Number of patients with low serum C peptide level was very small to give any valid conclusion regarding the association of serum C peptide level with renal parameters. Furthermore, a cross-sectional study design tends to leave uncertainty regarding the temporal sequence of exposure-outcome relations. Thus, confirming the relation with prospective longitudinal data would be valuable.

## Conclusion

The study has shown weak association of serum peptide level with microalbuminuria and creatinine clearance. Patients with low serum C peptide level may have increased risk of microalbuminuria. C-peptide replacement together with insulin administration may be beneficial in type 1 diabetes patients. Studies involving C-peptide administration of longer duration will be required to determine whether C-peptide may have a role in the prevention and treatment of diabetic nephropathy.
